# A reevaluation of the basal turtle *Indochelys spatulata* from the Early–Middle Jurassic (Toarcian–Aalenian) of India, with descriptions of new material

**DOI:** 10.7717/peerj.8542

**Published:** 2020-02-11

**Authors:** Walter G. Joyce, Saswati Bandyopadhyay

**Affiliations:** 1 Department of Geosciences, University of Fribourg, Fribourg, Switzerland; 2 Geological Studies Unit, Indian Statistical Institute, Kolkata, India

**Keywords:** Kota formation, Jurassica, Telengana state, Maharashtra state, Testudinata

## Abstract

**Background:**

*Indochelys spatulata* is an extinct turtle from the Early to Middle Jurassic Kota Formation of the Pranhita–Godavari Gondwana basin, India. The holotype and previously only known specimen is a partially eroded shell that had been collected near Kota village, north of Sironcha, in Maharashtra State. Phylogenetic analyses have consistently suggested placement at the base of the clade Mesochelydia.

**Methods:**

We here figure and describe the holotype of *Indochelys spatulata* and two new specimens, which were collected from the Kota Formation near Kistapur village, Telengana State, about 60 km NW from the type locality. We furthermore explore the relationships of this fossil turtle by updating its scoring based on all available material in the most recent analysis of basal turtle relationships.

**Results:**

The revision of the holotype of *Indochelys spatulata* provides minor adjustments to the morphology of this specimen, in particular recognition of a transverse break across the carapace, presence of only eight neurals, of which the eight is octagonal, and presence of a pathological element located between neurals VII and VIII. The new material provides new anatomical insights, in particular presence of a broad cervical, a vertebral V that inserts deeply into vertebral IV, narrow pleurals within increasingly short posteromedial contacts with the vertebrals towards the posterior, at least three pairs of musk duct foramina, and numerous insights into the morphology of the girdles and stylopodium. In combination, all material allows affirming the validity of *Indochelys spatulata* with confidence. The phylogenetic analysis affirms the placement of *Indochelys spatulata* as a basal mesochelydian, but cannot resolve its relationships relative to the roughly coeval *Condorchelys antiqua* and *Kayentachelys aprix*.

## Introduction

The fossil record of basal members of the clade Mesochelydia (sensu Joyce, 2017) is extremely sparse worldwide. Only five named species are currently recognized as valid: *Condorchelys antiqua* Sterli (2008) from the Early to Middle Jurassic of Argentina, *Eileanchelys waldmani* Anquetin et al. (2009) from the Middle Jurassic of the UK, *Heckerochelys romani* Sukhanov (2006) from the Middle Jurassic of European Russia, *Kayentachelys aprix* Gaffney et al. (1987) from the Early Jurassic of the USA, and, most notable for this contribution, *Indochelys spatulata* Datta et al. (2000) from the Early to Middle Jurassic of India (see Joyce, 2017 for recent summary). All species are either based on partial or crushed material that originates from poorly dated continental sediments. Many aspects of their morphology are therefore still poorly known and their phylogenetic relationships poorly resolved. As even minor insights into the anatomy of a taxon can have an impact on its phylogenetic relationships, we here take the opportunity to reassess the age and morphology of the holotype of *Indochelys spatula* and to provide the description of newly discovered material from the type formation.

## Geological Settings

**Figure 1:**
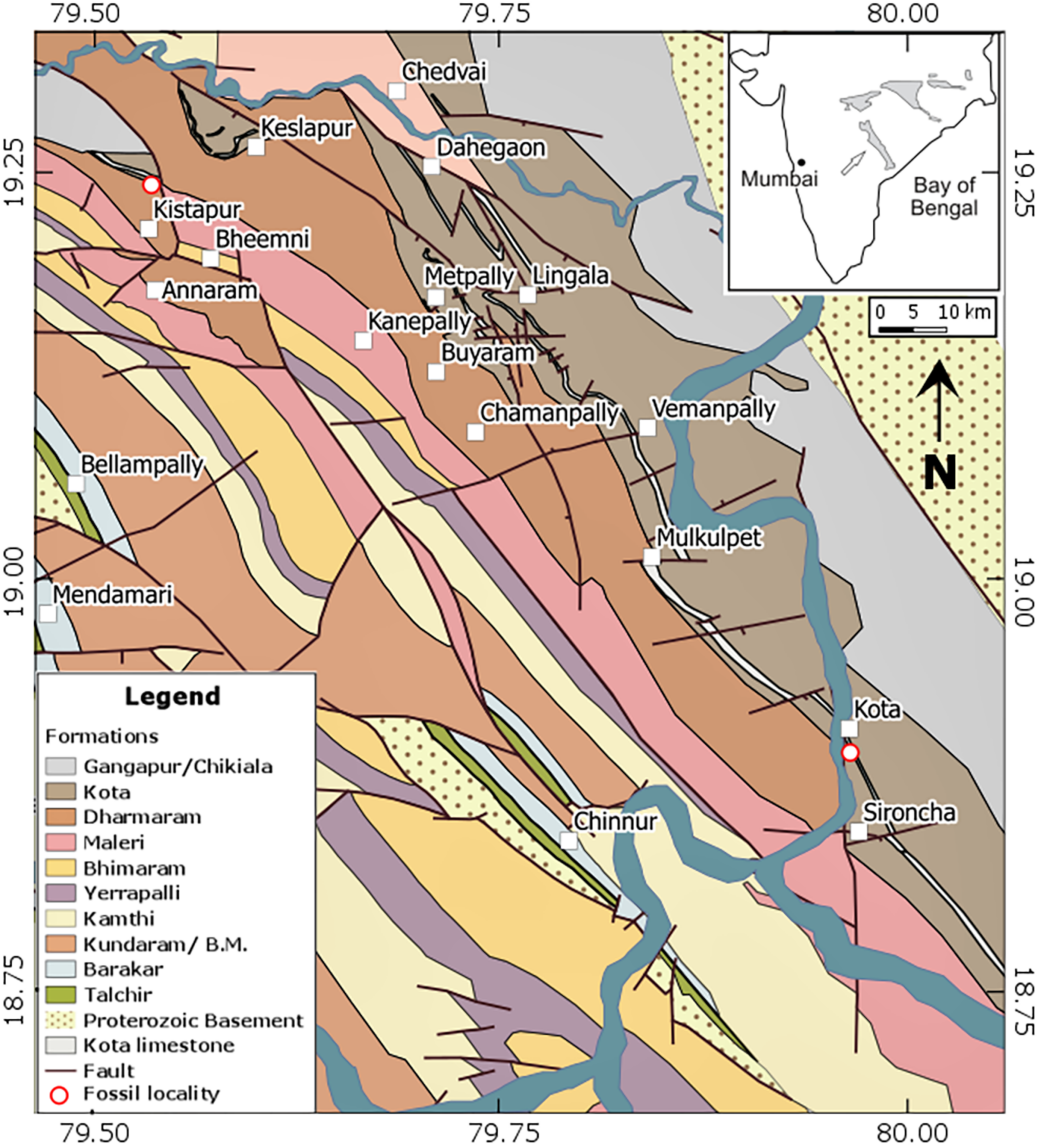
Detailed geological map of the Pranhita–Godavari basin in central India (modified after Kutty, Jain & Roy Chowdhury, 1987). The inset highlights the location of the Pranhita–Godavari basin in India. Red circles denote localities that yielded specimens of *Indochelys spatulata*, in particular the type locality near Kota village to the bottom right and the new locality near Kistapur village to the top left.

The Gondwana successions of India occur in several isolated basins in peninsular India such as the Pranhita–Godavari, Damodar, Satpura, and Son–Mahanadi basins (Pascoe, 1959; Robinson, 1967; Veevers & Tewari, 1995). *Indochelys spatulata* has so far only been recovered from the Early to Middle Jurassic Kota Formation of the Pranhita–Godavari (henceforth P–G) basin (Datta et al., 2000; Fig. 1). The Gondwana succession of the P–G basin occurs as a narrow, rectilinear outcrop trending NNW–SSE and is bordered on both sides by Proterozoic and/or Archaean rocks (Chakraborty, Mandal & Ghosh, 2003) and is overlain by the Deccan Trap basalt of Cretaceous to Palaeocene age (69–63 Ma; Pande, 2002). The overall dip of the succession is 5–12 degrees north and north-west with a general northward palaeocurrent direction (Sengupta, 1970). The Kota Formation in the Upper Gondwana of the P–G basin is about 500–600 m thick, showing a more or less uniform lithology throughout the valley (Goswami, Gierlowski-Kordesch & Ghosh, 2018). This formation overlies the fluvial deposits of the Dharmaram Formation and is unconformably overlain by the Gangapur Formation and is divided into two lithological units (Rudra, 1982). The 100 m thick lower unit is dominantly comprised of 20–30 m thick limestone beds followed upward by ferruginous mudstone, argillaceous and ferruginous sandstone, and calcareous sandstone (Goswami, Gierlowski-Kordesch & Ghosh, 2018). Rudra & Maulik (1994) suggested that a meandering river system deposited the Lower Kota, while a braided river system formed the upper part. The limestone facies was interpreted to be a freshwater carbonate deposit (Goswami, Gierlowski-Kordesch & Ghosh, 2018).

The Kota Formation is a rich storehouse of Early to Middle Jurassic vertebrate fauna that has been collected from two successive stratigraphic levels. From the mudstone dominated fluviatile lower unit, two sauropod dinosaurs, *Barapasaurus tagorei* (Jain et al., 1975, 1979; Bandyopadhyay et al., 2010) and *Kotasaurus yamanpalliensis* (Yadagiri, 1988, 2001) and some theropod teeth and ankylosaur bones (Galton, 2019) have been recovered. On the basis of isolated post canine teeth, a kuehneotherid (*Kotatherium haldanei*) (Datta, 1981) and a morganucodontid (*Indotherium pranhitai*) (Yadagiri, 1984) have also been identified. The limestone-dominated lacustrine deposit of the upper unit have yielded three semionontids (*Lepidotes deccanensis*, *Paradapedium egertoni*, and *Tetragonolepis oldhami*) (Jain, 1959, 1973, 1983), a pholidophorid (*Pholidophorous kingi*) (Yadagiri & Prasad, 1977), and a coelacanth (*Indocoelacanthus robustus*) (Jain, 1974a). Among the reptiles there are a pterosaur (*Campylognathoides indicus*) (Jain, 1974b), a mesosuchian crocodylomorph (Bandyopadhyay & Roychowdhury, 1996), and a turtle (*Indochelys spatulata*) (Datta et al., 2000), which is presented herein.

The micro-vertebrate assemblage (Yadagiri, 1986) in the upper unit comprises an elasmobranch (*Lissodus indicus*), a rhynchocephalian, and fragments of a pleurodont dentition, identified as *Paikasisaurus indicus*. Later, Prasad, Manhas & Arratia (2004) described two elasmobranchs? *Polyacrodus* sp. and *Lissodus indicus* along with some other ‘holosteans’. Among the lepidosaurs, two sphenodontians, *Rebbanasaurus jaini* and *Godavarisaurus lateefi*, and three dentary fragments and partial maxilla of a probable pleurodont lepidosauromorph similar to basal rhynchocephalians were described by Evans, Prasad & Manhas (2001). Subsequently, Evans, Prasad & Manhas (2002) described an acrodont lizard, *Bharatagama rebbanensis*, and two indeterminate agamid lizards. Among six described micromammals there are a docodont, *Gondtherium dattai* (Prasad, 2003) and a small lower molar of uncertain affinities, *Dyskritodon indicus* (Prasad & Manhas, 2002), two dryolestids and a probable ‘amphilestid’ *Paikasigudodon yadagiri* (Prasad & Manhas, 1997, 2002), a therian (*Trishulotherium kataensis*) (Yadagiri, 1984), and a holotherian (*Nakunodon paikasiensis*) (Yadagiri, 1985) of uncertain familial affinities (Averianov, 2002). Recently, an isolated upper premolar (P^4^) of a multituberculate, *Indobaatar zofiae*, has been reported from this horizon (Parmar, Prasad & Kumar, 2013).

The Kota Formation of the P–G basin has long been considered to be of Early Jurassic (Liassic) age on the basis of its fishes (King, 1881; Robinson, 1967). Analyzing the faunas of the lower and upper units of the Kota Formation and those of the underlying Dharmaram Formation and comparing them with faunas from coeval horizons, Bandyopadhyay & Roychowdhury (1996) and Bandyopadhyay & Sengupta (2006) suggested that the Lower Kota Formation has an age ranging from Sinemurian to Pliensbachian, while the age of the upper Kota is Toarcian and may even be extended to Middle Jurassic (?Aalenian).

## Materials

This study is based on three specimens referable to *Indochelys spatulata*: GSI 20380, the holotype, ISI R176, and ISI R177. The localities that yielded these specimens are discussed below (see “Systematic Paleontology”).

### GSI 20380

**Figure 2:**
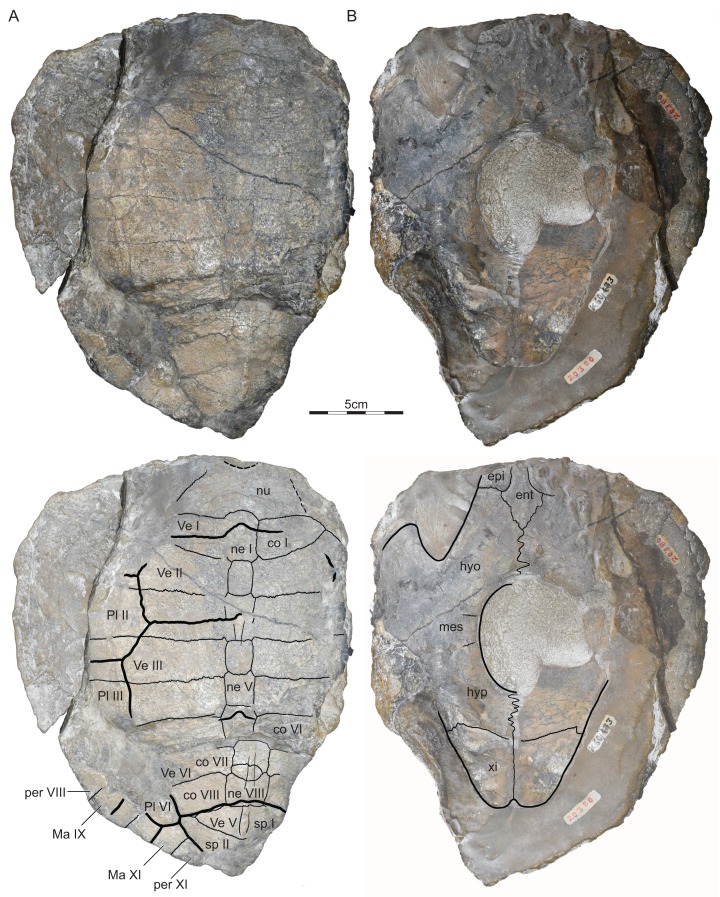
GSI 20380, *Indochelys spatulata*, holotype, Maharashtra, India, Kota Formation, Early–Middle Jurassic. Photographs and illustrations of eroded shell in (A) dorsal and (B) ventral view. co, costal; ent, entoplastron; epi, epiplastron; hyo, hyoplastron; hyp, hypoplastron; Ma, marginal scute; mes, mesoplastron; ne, neural; nu, nuchal; per, peripheral; Pl, pleural scute; sp, suprapygal; Ve, vertebral scute; xi, xiphiplastron.

The holotype once consisted of a complete shell, but erosion partially exposed the steinkern (Fig. 2). Only the internal imprints are therefore available for much of the plastron and the peripheral series. The specimen furthermore shows disarticulation at the confluence of the costal VI/VII suture and the vertebral III/IV sulcus, which likely occurred after death, but before final burial.

### ISI R176

**Figure 3:**
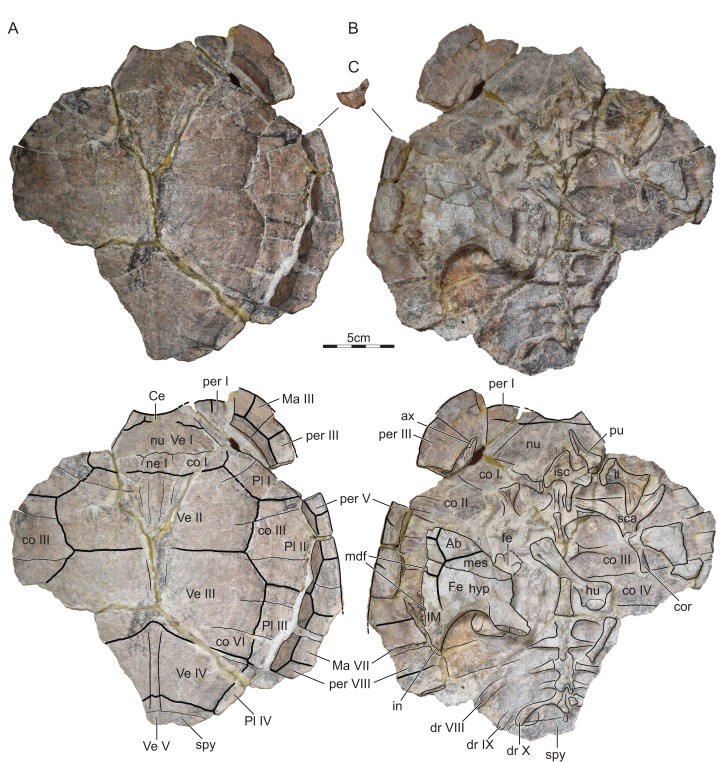
ISI R176, *Indochelys spatulata*, Telangana, India, Kota Formation, Early–Middle Jurassic. Photographs and illustrations of partial skeleton in (A) dorsal and (B) ventral view and photograph of right peripheral V in anterior view with the dorsal side oriented towards the top (C). Ab, abdominal scute; ax, axillary buttress; Ce, cervical scute; co, costal; cor, coracoid; dr, dorsal rib; fe, femur; Fe, femoral scute; hu, humerus; hyp, hypoplastron; il, ilium; IM, inframarginal scute; in, inguinal buttress; isc, ischium; Ma, marginal scute; mdf, musk duct foramen; mes, mesoplastron; ne, neural; nu, nuchal; per, peripheral; Pl, pleural scute; pu, pubis; sca, scapula; sp, suprapygal; Ve, vertebral scute.

This specimen is a partial skeleton that includes much of the carapace, some plastral fragments, numerous long bones, and elements of the girdles (Fig. 3). All elements show signs of disarticulation, with the exception of the central portions of the carapace, which are held together by partial synostosis.

### ISI R177

**Figure 4:**
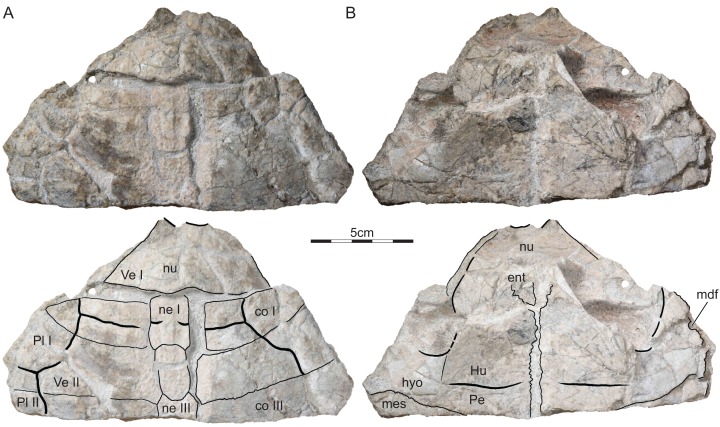
ISI R177, *Indochelys spatulata*, Telangana, India, Kota Formation, Early–Middle Jurassic. Photographs and illustrations of partial shell in (A) dorsal and (B) ventral view. co, costal; ent, entoplastron; Hu, humeral scute; hyo, hyoplastron; mdf, musk duct foramen; mes, mesoplastron; ne, neural; nu, nuchal; Pe, pectoral scute; Pl, pleural scute; Ve, vertebral scute.

This specimen consists of the anterior third of a shell (Fig. 4). Associated long bones and pelvic girdle elements are preserved within the shell, but are not figured, as they are only partially prepared and reveal no anatomical details deemed important. The specimen shows signs of disarticulation that occurred prior to final burial and numerous break that occurred later after the consolidation of the matrix.

We herein mostly compare *Indochelys spatulata* to the postcranial material of other basal mesochelydians, in particular *Condorchelys antiqua* Sterli (2008) (as described by Sterli & de la Fuente (2010) and Sterli, de la Fuente & Rougier (2018)), *Eileanchelys waldmani* Anquetin et al. (2009) (as described by Anquetin (2010)), *Heckerochelys romani* Sukhanov (2006) (as described by Sukhanov (2006); W.G. Joyce, 2019, personal observations of material at PIN), and *Kayentachelys aprix* Gaffney et al. (1987) (W.G. Joyce, 2019, personal observations of material at MCZ, MNA, TMM and UCMP).

## Systematic Paleontology

TESTUDINATA Batsch, 1788

MESOCHELYDIA Joyce, 2017

***Indochelys spatulata* Datta et al., 2000**

Holotype.—GSI 20380, a partial shell (Datta et al., 2000, Figs. 3 and 4; Fig. 2).

Type locality and horizon.—Near Kota village, north of Sironcha, Maharashtra State, India (Fig. 1); Kota Formation, Early to Middle Jurassic (Toarcian–Aalenian).

Referred material.—ISI R176, a partial shell (Fig. 3) and ISI R177, the anterior half of a shell (Fig. 4), collected 3 km NNE of Kistapur village (19°15′01.86″, 79°32′05.23″), Telengana State (formerly Andhra Pradesh), Kota Formation, Early to Middle Jurassic (Toarcian–Aalenian).

Emended diagnosis.—*Indochelys spatulata* can be diagnosed as a representative of Testudinata by the presence of a fully formed shell and as a representative of Mesochelydia by the presence of only eleven pairs of peripherals, absence of supramarginal scutes, and the reduction of the coracoid foramen. Among basal mesochelydians, *Indochelys spatulata* can be differentiated by having a distinct nuchal notch (closest to *Kayentachelys aprix*), a neural formula of 6p–4–8–4–6a–6a–6a–6a, lacking a contact between costal I and peripheral III, presence of a broad suprapygal II that blocks contact of costal VIII with peripheral XI (also in *Eileanchelys waldmani*), anteriorly shifted posterior pleural resulting in a short contact only between vertebral IV and pleural III (closest to *Condorchelys antiqua*), a chevron-shaped vertebral IV (closest to *Condorchelys antiqua*), and a free dorsal rib X.

## Description

Shell.—The full outline of the shell is not preserved in any specimen (Figs. 2–4). The presence of a distinct nuchal notch is suggested by the imprint of the nuchal in the holotype (Fig. 2) and confirmed by ISI R176 (Fig. 3). Although the adjacent peripherals are damaged in the latter specimen, it seems clear that the nuchal notch was mostly framed by the nuchal and that peripherals I only contributed little to this feature. The remaining peripherals suggest that the shell was overall rounded. All specimens are flattened. However, the three-dimensionally preserved bridge peripherals of ISI R176 (Fig. 3C) suggest that the shell was moderately domed in life. A faint midline keel is apparent in the holotype (Fig. 2) and ISI R176 (Fig. 3), which is more pronounced at the anterior margins of the vertebrals. The surface of the carapace lacks a notable microtexture, but the relatively deeply incised sulci combined with the slightly domed vertebral and pleural scutes give the shell a quilted appearance (Fig. 3).

The outline of the shell of *Indochelys spatulata* generally resembles that of other basal mesochelydians, but variation is apparent to the depth of the nuchal notch. It is best developed in *Indochelys spatulata*, closely followed by *Kayentachelys aprix*. The notch is only subtly developed in *Eileanchelys waldmani* and *Heckerochelys romani*. This aspect of the shell is not known for *Condorchelys antiqua*.

Nuchal.—The nuchal is present in all three available specimens, but it is best preserved in ISI R176 (Figs. 2–4). The nuchal has a trapezoidal outline and forms a distinct nuchal notch. The nuchal has an oblique anterolateral contact with peripheral I, a straight posterior contacts with costal I, and forms a deep median concavity that holds neural I. Datta et al. (2000) identified a medially located triangular notch in the holotype as a cervical scute, but our own observations of the holotype suggest that this is damage to the surface of this specimen (Fig. 2). A slightly raised median pedestal partially hidden by the ischium is apparent on the visceral side of ISI R176 (Fig. 3).

The nuchal of *Indochelys spatulata* closely resembles that of all other basal mesochelydians in its relative size, shape, and contacts. Variation to the depth of the nuchal notch is outlined above.

Neurals.—The neural series is best preserved in the holotype (Fig. 2). In this specimen, the series consists of eight regular neurals and a supernumerary element that is squeezed between neurals VII and VIII, not nine full neurals as reported by Datta et al. (2000). The neurals overall have a similar width, but notable variation is apparent to their shape and anteroposterior length. Neural I is the longest element in the series. It is a hexagonal element with a protruding anterior contact with the nuchal, straight lateral contacts with costals I, short posterolateral contacts with costals II, and a straight posterior contact with neural II. This morphology is confirmed by ISI R176 and ISI R177. Neural II is much shorter than neural I and has a rectangular outline. Neural III is slightly shorter than neural I, but is octagonal, not hexagonal, as suggested by Datta et al. (2000). This element therefore has lateral contacts with costals II–IV. Neural IV resembles neural II by being shorter than neurals I and III and by having a rectangular outline. Neurals V–VIII overall resemble one another by being hexagonal and by having similar dimensions. Datta et al. (2000) reported the presence of a full neural between neurals VII and VIII, but we reinterpret this as an asymmetric, supernumerary element that is located between the left contact of neurals VII and VIII. Neural VIII has a straight posterior contact with suprapygal I. The full neural series is present in ISI R176, but the elements that make it up cannot be discerned, as the central part of the carapace is fused.

All basal mesochelydians have a contiguous series of neural elements that hinder a medial contact of the costal series, but variation is apparent in the number and shape of elements. The neural series of *Kayentachelys aprix* resembles that of *Indochelys spatulata* in that neurals I–V form the formula 6p–4–8–4. However, the posterior four neurals of *Kayentachelys aprix* are shifted to the anterior, giving them a more symmetric, hexagonal outline, while the posterior four neurals of *Indochelys spatulata* are hexagonal with short anterolateral sides. The neural formula of *Heckerochelys romani* is 6p–6p–4–6–?–?–?–?, that of *Eileanchelys waldmani* 6p–6p–6p–4–6p–6p–4–4, and that of *Condorchelys antiqua* 6p–4–6a–6a–6a–4–4–4.

Costals.—The costals are best preserved in the holotype (Fig. 2). The carapace includes eight pairs of costals that lack a midline contact. Carapacial fontanelles are absent. As a general trend, the costal are rectangular elements that decrease in anteroposterior length from front to back. The notable exception is costal II, which greatly increases in anteroposterior length distally at the expense of costal I, which decreases distally instead. As a result, costal I is much narrower mediolaterally than the subsequent costals. The medial contacts are described above (see “Neurals”). The distal contacts of the anterior costals are unclear, as the peripherals are not preserved in the holotype (Fig. 2) and disarticulated in ISI R176 (Fig. 3). However, costal I certainly contacted peripheral I, likely contacted part of peripheral II, but certainly did not contact peripheral III. Costal II likely contacted peripheral II, but otherwise contacted peripherals III and IV and the anterior quarter of peripheral V, as apparent from ISI R176. The rib of costals III–VI distally insert at the junction of peripherals IV–VIII and each costal therefore contacts two peripherals. The lateral contacts of costals VII and VIII are not preserved, but they likely contacted peripherals VIII–X, but not peripheral XI, due to the presence of extremely broad suprapygals.

Variation is apparent among basal mesochelydians in regards to the relative size of costal I and the associated distal expansion of costal II. Costal I is smallest in *Indochelys spatulata* and a distal contact with peripheral III is clearly absent. This element is slightly larger in *Kayentachelys aprix* and a point contact is apparent with peripheral III. The element is larger yet again in *Condorchelys antiqua* and *Eileanchelys waldmani* and a clear contact is present with peripheral III. In all taxa, costal II expands distally at the expense of costal I. The only exception is *Heckerochelys romani*, which has rectangular costals I and II. Apparent variation in regards to the posterior contacts of costal VIII are discussed below (see “Suprapygals and Pygal”).

Peripherals.—*Indochelys spatulata* possesses eleven pairs of peripherals, of which peripherals VIII–XI can be identified in the holotype (Fig. 2) and peripherals I–III and V–VIII are preserved in ISI R176 (Fig. 3). The outer margins of peripherals I–VII are partially preserved in ISI R176, suggesting that the anterior half of the shell is smooth and lacks serrations. The margins of all posterior peripherals are damaged and the outline of this part of the shell is therefore unknown. The medial contacts of the peripherals with the costals are outlined above (see “Costals”). As costals II–VI insert between peripherals, the costoperipheral suture forms a zig-zag pattern. This pattern is highly apparent in *Kayentachelys aprix*, likely present in *Condorchelys antiqua*, but cannot be evaluated well for all other basal mesochelydians.

The axillary buttress contacts the posterior quarter of peripheral II, the inguinal buttress the anterior third of peripheral VIII. Peripherals VI–VII are therefore fully formed bridge peripherals with a Y-shaped cross-section (Fig. 3C). The wide-angle apparent between the dorsal and ventral branches of the bridge peripherals, most apparent in the anterior view of peripheral V (Fig. 3C), suggests that *Indochelys spatulata* was moderately domed. In all regards, these observations correspond to those that can be made on material of *Condorchelys antiqua* and *Kayentachelys aprix*, but cannot be evaluated for other basal mesochelydians.

Suprapygals and pygal.—The holotype of *Indochelys spatulata* possesses two suprapygals and leaves room for a pygal (Fig. 2). ISI R176 only preserves the anterior part of suprapygal I (Fig. 3). Suprapygal I is a pentagonal element that is twice as broad as neural VIII. It contacts neural VIII anteriorly, costal VIII anterolaterally, and suprapygal II posteriorly at an angled contact. In contrast to Datta et al. (2000) we are only able to reconstruct the anterior and left lateral portions of suprapygal II. From what can be discerned, suprapygal II is significantly broader than suprapygal I and boomerang-shaped. It has an angled, concave anterior contact with suprapygal I, a short anterolateral contact with costal VIII, and short posterolateral contacts with peripherals X and XI, which coincide with the sulcus between marginal XI and vertebral V. We cannot discern the likely posterior contacts of suprapygal II with the medial parts of peripheral XI and the pygal. In all cases, suprapygal I is narrower than suprapygal II.

The suprapygals of *Indochelys spatulata* resembles those of *Eileanchelys waldmani* the most, by being much broader than the neural series and by blocking costal VIII from contacting peripheral XI. The suprapygals of *Heckerochelys romani* are narrower and a point contact is therefore present between costal VIII and peripheral XI. The suprapygals of *Condorchelys antiqua* and *Kayentachelys aprix* are even narrower and a clear contact is present between costal VIII and peripheral XI.

Carapacial scutes.—The carapace of *Indochelys spatulata* is covered by a cervical, five vertebrals, four pairs of pleurals, and twelve pairs of marginals, the plesiomorphic condition for Mesochelydia (Figs. 2–4). Datta et al. (2000) reported the presence of a triangular cervical that inserts deeply into vertebral I, but we conclude that the cervical region is damaged in this specimen. Although the sulci are faint, ISI R176 documents a broad cervical instead that covers the anterior half of the anterior nuchal margin, but only a quarter of the nuchal depth. It contacts marginal I laterally and vertebral I posteriorly. The cervical is broadly comparable to those of all other basal mesochelydians.

The series of very broad vertebrals is jointly documented by the holotype and ISI R176 (Figs. 2 and 3). Vertebral I is a polygonal element that is about twice as broad as long. The lateral contacts are not preserved in any specimen, but the preserved portions of the shell suggest an anterior contact with the cervical, a broad anterolateral contact with marginal I, a lateral contact with pleural I, and a posterior contact with vertebral II. An additional, anterolateral contact with marginal II is possible, but would be small, if present. Vertebral II is a hexagonal element that is about 40% broader than vertebral I. It laterally contacts pleurals I and II. The anterior contact with vertebral I is concave, but the posterior contact with vertebral III straight. Vertebral III is a broad, hexagonal element that is about 50% broader than vertebral I. The anterolateral contact with pleural II is shorter than the posterolateral contact with pleural III. The anterior contact with vertebral II is straight, but the posterior contact with vertebral IV deeply concave. Vertebral IV is an unusual, chevron-shaped element that anteriorly inserts deeply into vertebral III, but posteriorly receives vertebral V. The short anterolateral contact with pleural III and the elongate posterolateral contact with IV together form a straight line. Only the anterior two-thirds of vertebral V are documented. This element is the narrowest element in the series. It anteriorly inserts deeply in vertebral IV, laterally has a short contact with pleural IV, posterolaterally has a broad contact with marginal XI, and likely had a broad posterior contact with marginal XII. The intervertebral sulci are located at the anterior third of neural I, the middle of neural III, the anterior third of vertebral VI, and the posterior margin of neural VIII.

The posterior pleurals of *Indochelys spatulata* are shifted to the anterior relative to the vertebrals, resulting in a reduced anterolateral contact of vertebral III with pleural II and a notably short anterolateral contact of vertebral IV with pleural III. These contacts are symmetric in other basal mesochelydians, with the exception of *Condorchelys antiqua*, which shows a reduced contact of vertebral III with pleural II. Variation is apparent among basal mesochelydians to the degree to which the chevron shape is developed for vertebral IV. It is fully absent in *Eileanchelys waldmani*, slightly developed in *Kayentachelys aprix*, even better developed in *Condorchelys antiqua*, but the most extreme by far in *Indochelys spatulata*.

The pleurals are much narrower than the vertebrals. In addition to the vertebral contacts described above, pleural I contacts marginals II–V, pleural II contacts marginals V–VII, pleural III contacts marginals VII–IX, and pleural IV contacts marginals IX–XI. A contact may be present between pleural I and marginal I, but it is more likely that this contact was hindered by the contact of vertebral I with marginal II. These observations broadly agree with those made for other basal mesochelydians.

Although the posterior margin of the shell is not known, it is highly likely that 12 pairs of marginals are present. The medial contacts with the pleurals and vertebrals are described above. Marginals that contact a single element medially have notably low, straight sulci. Those that contact two elements, possess high triangular sulci. The pleural/marginal sulci nevertheless are always restricted to the peripherals. These observations, too, broadly agree with those made for other basal mesochelydians.

Plastral bones.—The plastron of *Indochelys spatulata* is poorly known, as the plastron of the holotype (Fig. 2) only consists of highly eroded bone or the internal imprint of the plastron and those of ISI R176 and ISI R177 are only fragments (Figs. 2 and 3).

The internal imprint preserved in the holotype suggests that the plastron of *Indochelys spatulata* consists of an entoplastron and paired epi-, hyo-, meso-, hypo-, and xiphiplastra. The probably similarly sized anterior and posterior plastral lobes gently taper towards their tips. A small, but distinct anal notch is present. In contrast to Datta et al. (2000), we conclude that the holotype possesses an elongate central plastral fontanelle that hinders the mesoplastra from contacting one another along the midline.

Datta et al. (2000) documented the anterior margin of the plastron, but we note that the preserved margin of the internal imprint must not necessarily represent the anterior margin of the plastron, although the implied dimensions are reasonable. At the very least, it is apparent that the entoplastron is a dagger-shaped element with an elongate anterior process that likely hindered the blocky epiplastra from contacting one another along the midline, at least along the visceral side of the plastron. The length of the posterior entoplastral process is unknown and the presence of epiplastral processes unclear. The distal end of the axillary buttress inserts into the posterior quarter of peripheral II, while the posterior buttress inserts into the anterior third of peripheral VIII, as suggested by ISI R176 (Fig. 3). At least three musk duct foramina are present, one at the contact of the hyoplastron with peripheral IV, as suggested by ISI R177 (Fig. 4) and two at the contact of the hypoplastron with peripherals VI and VII, as suggested by ISI R176 (Fig. 3). Additional musk duct foramina that might be present between the mesoplastron and peripheral V cannot be discerned, but preservation is suboptimal in these parts of the available material.

The little available morphology is overall comparable with that of other basal mesochelydians. An elongate anterior plastral process that separates the epiplastra, however, is only known to occur in *Condorchelys antiqua* and *Kayentachelys aprix*, but not in *Eileanchelys waldmani*. The presence of central plastral fontanelles is variable as well, but additional material is needed to clarify if this is an artifact of uneven ontogenetic sampling. The two musk duct foramina observed in *Indochelys spatulata* correspond in their placement to those of *Condorchelys antiqua* and *Kayentachelys aprix*, but the latter is known to have at least three musk duct foramina, the latter at least four.

Plastral scutes.—Only five scutes are partially preserved in ISI R176 and ISI R177, which we interpret as the abdominals, femorals, and inframarginals II–IV. The abdominal/femoral sulcus is located on the hyoplastron near its suture with the mesoplastron. It laterally contacts inframarginal III. The three inframarginals are blocky elements located at the lateral margin of the plastron that fully block contact of the carapacial scutes with the central plastral scutes. Though limited, the available morphology closely approximates that of other basal mesochelydians.

Dorsal column.—The dorsal column and the associated ribs are best preserved in ISI R176, but it is difficult to discern many details with confidence, as parts of the column are crushed, disarticulated, or covered from view by other elements. The dorsal centra are relatively flat and have broad contacts with one another. A disarticulated anterior centrum highlights that the vertebrae of the dorsal column were not fused to one another, at least in the anterior portion of the shell. The dorsal rib heads are elongate and have broad contacts with the vertebrae. This suggests the presence of a well-developed costovertebral tunnel. Among basal mesochelydians, the dorsal column has only been described for *Condorchelys antiqua*, but poor preservation in both taxa makes meaningful comparison taxing.

The first dorsal rib is partially preserved in ISI R177 (not figured). Its proximal head is flattened and oriented vertically, but its distal extent is unclear. The ribs of costals I–VI (i.e. dorsal ribs II–VII), as seen in ISI R176 are relatively broad distally and mostly oriented laterally, but the anterior two are slightly oriented to the anterior, the posterior two slightly to the posterior, too. The proximal portions of costal ribs VII and VIII (e.g. dorsal ribs VIII and IX) are oriented laterally as well, but the distal halves expand only little and curve strongly to the posterior to form 45- and 90-degree arcs, respectively. Dorsal rib X is shorter than dorsal rib IX, but is also strongly curved towards the posterior, but only forms a 30-degree arc. A gap between dorsal rib X and the shell suggests that it was only loosely attached to the shell at best. The orientation of the dorsal ribs fully overlaps with those of *Kayentachelys aprix*, the only other mesochelydian for which this part of the skeleton is known, but dorsal rib X is tightly fused with the shell, in *Kayentachelys aprix*, as in the majority of turtles. An unattached dorsal rib X has otherwise been reported for the basal perichelydian *Naomichelys speciosa* (Joyce, Sterli & Chapman, 2014).

Girdles.—The left scapulocoracoid and the right pelvis are associated with ISI R176 (Fig. 3). The scapulocoracoid is disarticulated at the glenoid, much of the coracoid is damaged, and parts of this structure are covered from view by other elements. The scapular process and the glenoid process jointly form a distinct neck above the glenoid. The scapular process is clearly longer than the glenoid, but its full length cannot be ascertained, as it is distally covered by the ischium. The distal end of the acromion is slightly recurved ventrally. The scapula possesses an extensive webbing of bone that spans between the scapular process and the acromion. As the element is viewed from medial, we are unable to clarify the additional presence of additional bony webbing between the scapular process and the glenoid and between the acromion and the glenoid. The coracoid is too damaged to allow drawing anatomical conclusions, beyond the obvious absence of a formed coracoid foramen. The scapula of *Indochelys spatulata* has a particularly extensive webbing between the scapular and acromion processes. In this regard, *Indochelys spatulata* more closely resembles more basal turtles, such as *Proganochelys quenstedti* (Gaffney, 1990) and *Proterochersis* spp. (Joyce, Schoch & Lyson, 2013; Szczygielski & Sulej, 2016), than other basal mesochelydians. *Indochelys spatulata* furthermore resembles more basal turtles by having a recurved acromion, though in a different direction. *Indochelys spatulata* differs from more basal turtles, but resembles all other basal mesochelydians, by having a strap-like coracoid and therefore lacking a coracoid foramen.

The right pelvis is disarticulated. The ilium and pubis are preserved in medial view, but the ischium disarticulated relatively to the rest and now can be viewed in dorsal view. The acetabulum therefore appears to not have been fused. The ilium has a robust shaft and a dorsal fan that mostly expands to the posterior. The pubis is too damaged to allow assessing the presence and shape of a lateral process or development of an epipubic process. The pubis forms a broad medial plate that suggests that the midline contact of the pubes was relatively extensive. The metischial process is elongate and protrudes posteriorly far beyond the level of the main plate of the ischium. It is not clear if the thyroid fenestrae have coalesced, but the extent of the ischial and pubic plates suggests that they were near separate to fully separate from one another. Though comparisons are limited, the pelvic anatomy of *Indochelys spatulata* resembles that of other basal mesochelydians, in particular *Condorchelys antiqua* and *Kayentachelys aprix*. The outwardly curved posterior margins of the medial ischial plate is consistent with the presence of a supernumerary bone, as seen in *Condorchelys antiqua*, which may represent a remnant hypoischium (Sterli, de la Fuente & Rougier, 2018).

Limbs.—The right humerus is preserved in dorsal view and the left femur in ventral view in ISI R176 (Fig. 3). The humerus head is slightly ovoid, but it is unclear if a shoulder is developed. The medial process splays slightly to the side, while the lateral process is short and tucked just to the side of the head. The shaft is slim and slightly sinusoidal. The ectepicondylar groove is enclosed into a canal. The head of the femur is damaged, but imprints in the matrix suggest that the femur was about the same length as the humerus and that the trochanters form a narrow intertrochanteric fossa. Though comparisons are limited, the morphology of the stylopodium of *Indochelys spatulata* generally resembles that of other basal mesochelydians.

## Phylogenetic Analysis

To investigate the impact of our novel observations, we updated the scoring of *Indochelys spatulata* in the matrix of Sterli, de la Fuente & Rougier (2018), the most recent analysis of basal turtle relationships. Only a single character demanded correction (1, not 0, for character 131), but 29 additional characters could be scored for the first time (e.g. characters 127, 128, 133, 139, 145, 147, 153, 159, 161, 163, 164, 179, 181, 182, 202, 203, 204, 205, 207, 217, 220, 221, 223, 224, 225, 226, 230, 234, 235). The scoring of *Indochelys spatulata* could thereby almost be doubled and the amount of missing data reduced from 84.6% to 72.3%, including non-applicable scorings (see File S1).

**Figure 5:**
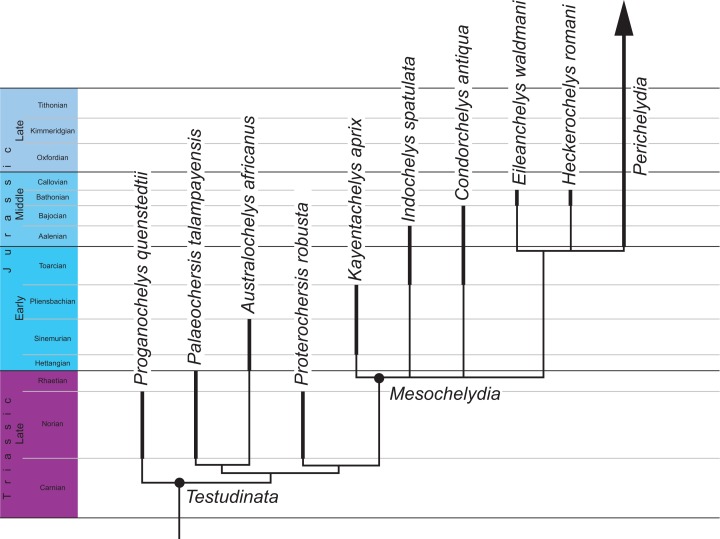
Phylogenetic hypothesis of basal turtles. Time-calibrated strict consensus cladogram retrieved from the phylogenetic. Dark lines highlight the known temporal distribution of a species.

The matrix was subjected to a parsimony analysis using the software TNT (Goloboff, Farris & Nixon, 2008). Following Sterli, de la Fuente & Rougier (2018) with the 28 characters that form morphoclines were run ordered and no backbone was implemented. However, following the recent recommendations of Goloboff, Torres & Arias (2018), light implied weighting was implemented with a *k* value of only 12. The matrix was subjected to 1,000 replicates of random addition sequences followed by a second round of tree bisection-reconnection. The best score was hit 54 times. An extract of the strict consensus of the 30 most parsimonious solution (see File S1 for all trees) with a score of 35.25875 is provided in Fig. 5.

## Discussion

The holotype of *Indochelys spatulata*, GSI 20380, was collected from the Kota Formation near the village of Kota, Maharashtra, India. Our interpretation of the morphology of this specimen generally resembles that of Datta et al. (2000), but nuanced differences are apparent. In addition to us having less confidence about the outline of the carapace and plastron, we recognize disarticulation and displacement of the posterior portion of the carapace, presence of an octagonal, not hexagonal neural VIII, presence of an asymmetric, supernumerary neural, not a ninth neural, development of a short, but broad cervical, not a deep, triangular cervical, and presence of a central plastral fontanelle.

The new specimens presented herein, ISI R176 and ISI R177, were collected from the Kota Formation as well, but near the village of Kistapur, Telangana, India, across the river, about 58.5 km NW from the type locality. The new specimens resemble the holotype in overall shape and size, presence of a distinct nuchal notch, a low midline keel, short costal I, a laterally expanded costal II, broad vertebrals with asymmetric lateral sides, a hexagonal neural I, a square neural II. As all specimens originate from the same layer within the Kota Formation and overlap in their morphology, we confidently refer all to *Indochelys spatulata*.

The new material *Indochelys spatulata* provides much additional morphological information from the peripheral series, the carapacial scutes, the bridge, the visceral anatomy of the carapace, the girdles, and the stylopodium. *Indochelys spatulata* shows striking similarities with the near-coeval basal mesochelydians *Condorchelys antiqua* from Chubut, Argentina and *Kayentachelys aprix* from Arizona, USA in overall shape and size, the morphology of the girdles and limbs, and the arrangement and contacts of the bones and scutes. We are nevertheless able to more rigorously diagnose *Indochelys spatulata* relative to other basal mesochelydians with an extended series of characters, in particular the relatively deep nuchal notch, the unique neural formula, a short costal I that lacks contact with peripheral III, presence of a broad suprapygal II that blocks contact of costal VIII with peripheral XI, anteriorly shifted pleurals III and IV resulting in a point contact between vertebral IV and pleural III, a chevron shaped vertebral IV, and a particularly well-developed bone web between the scapular and acromion processes of the scapula.

To investigate the impact of our new anatomical insights, we modified the scoring of *Indochelys spatulata* in the character/taxon matrix of Sterli, de la Fuente & Rougier (2018), mostly by scoring *Indochelys spatulata* for 29 characters that had previously be scored as unknown. Given the great similarities between *Indochelys spatulata*, *Condorchelys antiqua*, and *Kayentachelys aprix*, it is not surprising that our phylogenetic analysis places these three turtles in an unresolved polytomy at the base of Mesochelydia (Fig. 5), which fully overlaps with the conclusions of Sterli, de la Fuente & Rougier (2018). This result is somewhat dispiriting, as the substantial reduction in missing data did not provide greater resolution, but also highlights the morphological homogeneity of basal mesochelydians. Considering that *Condorchelys antiqua*, *Indochelys spatulata*, and *Kayentachelys aprix* originate from localities positioned at the opposite ends of what used to be Pangea, this morphology homogeneity is nevertheless notable, as it highlights the faunal homogeneity that still existed across this continent prior to its break-up in the Middle Jurassic (Joyce et al., 2016).

## Conclusions

A revision of the holotype, in combination with new material from the type stratum, confirms that the Early to Middle Jurassic turtle *Indochelys spatulata* from the Kota Formation of India is a valid taxon. An updated phylogeny of basal turtles affirms its position at the base of Mesochelydia.

## Supplemental Information

10.7717/peerj.8542/supp-1 Supplemental Information 1 Character taxon matrix used in phylogenetic analysis, including full character list and character state definitions.

10.7717/peerj.8542/supp-2 Supplemental Information 2 Tree file containing the 30 most parsimonious solutions obtained by the phylogenetic analysis.

## Institutional Abbreviations

GSI: Geological Survey of India, Kolkata, India
ISI: Indian Statistical Institute, Kolkata, India
MCZ: Museum of Comparative Zoology, Cambridge, Massachusetts, USA
MNA: Museum of Northern Arizona, Flagstaff, Arizona, USA
TMM: Texas Memorial Museum, Austin, Texas, USA
PIN: Paleontological Institute of the Russian Academy of Sciences, Moscow, Russia
UCMP: University of California Museum of Paleontology, Berkeley, California, USA

## Additional Information and Declarations

### Data Availability

The following information was supplied regarding data availability: All specimens described herein are available for study at the Geological Survey of India, Kolkata, India (GSI), and the Indian Statistical Institute, Kolkata, India (ISI): catalogue numbers GSI 20380, ISI R176, and ISI R177. Character taxon matrix is available in the Supplemental Files.
